# On the Annotation of Health Care Pathways to Allow the Application of Care-Plans That Generate Data for Multiple Purposes

**DOI:** 10.3389/fdgth.2021.688218

**Published:** 2021-09-08

**Authors:** Martin Ingvar, Mathias C. Blom, Casper Winsnes, Greg Robinson, Lowie Vanfleteren, Stan Huff

**Affiliations:** ^1^Department of Clinical Neuroscience, Karolinska Institutet, Solna, Sweden; ^2^Department of Clinical Neuroradiology, Karolinska University Hospital, Stockholm, Sweden; ^3^The Boston Consulting Group, Boston, MA, United States; ^4^Frisq AB, Stockholm, Sweden; ^5^International Consortium for Health Outcomes Measurement, Boston, MA, United States; ^6^University of Gothenburg and Sahlgrenska University Hospital, Gothenburg, Sweden; ^7^Department of Biomedical Informatics, Intermountain Health Care, University of Utah, Salt Lake City, UT, United States

**Keywords:** care pathway, interoperability, outcomes - health care, machine learning, anonymization

## Abstract

**Objectives:** Procedural interoperability in health care requires information support and monitoring of a common work practice. Our aim was to devise an information model for a complete annotation of actions in clinical pathways that allow use of multiple plans concomitantly as several partial processes underlie any composite clinical process.

**Materials and Methods:** The development of the information model was based on the integration of a defined protocol for clinical interoperability in the care of patients with chronic obstructive pulmonary disease and an observational study protocol for cohort characterization at the group level. In the clinical process patient reported outcome measures were included.

**Results:** The clinical protocol and the observation study protocol were developed on the clinical level and a single plan definition was developed by merging of the protocols. The information model and a common data model that had been developed for care pathways was successfully implemented and data for the medical records and the observational study could be extracted independently. The interprofessional process support improved the communication between the stakeholders (health care professionals, clinical scientists and providers).

**Discussion:** We successfully merged the processes and had a functionally successful pilot demonstrating a seamless appearance for the health care professionals, while at the same time it was possible to generate data that could serve quality registries and clinical research. The adopted data model was initially tested and hereby published to the public domain.

**Conclusion:** The use of a patient centered information model and data annotation focused on the care pathway simplifies the annotation of data for different purposes and supports sharing of knowledge along the patient care path.

## Background

The delivery of patient-centered care in the context of increasing multimorbidity and medical complexity cannot be sustained by traditional healthcare systems (1). New approaches to health informatics are required to enable integration of health care processes between providers and healthcare settings. Future general health services need support for complex processes that are continuous across boundaries between providers while at the same time involving the patient (2). While the interoperability of healthcare data is already widely recognized as a key prerequisite for scaling and validating the use of artificial intelligence and advanced analytics applications in healthcare (3), interoperability is equally crucial to any scenario where qualified planning is implemented in the workflow of health information systems across multiple institutions (4). Without semantic interoperability, the flags and triggers necessary to identify who qualifies for which care plan and/or intervention, will not be reliable; thus potentially subjecting individual patients to care that is not beneficial or that may be harmful (5). Also, health care without tools to support standard operating procedures will function without procedural interoperability and the collected data will not have consistent semantic underpinnings. Thus, information systems that are not designed for information interoperability cannot support procedural interoperability nor generate data suitable for quality benchmarking or clinical research.

The urgent need for health care transformation can only be met through the development of suitable systems for information support. Systems that can deliver secure healthcare, fair revenue sharing between providers, quality control and promotion of systems learning, should ideally support a common documentation of the care pathway across the full care cycle that is shared among all stakeholders (6). A gradual move from information support that records single events to models that capture context is needed; for example, determination of whether and when a patient is enrolled in an orchestrated care plan requires a trans-professional agreement on the decision structure, which remains a challenging task. Reliable capture of context will also become increasingly important in mitigating biased healthcare decisions, as clinical decision support systems and related applications gain in popularity and start impact decisions at scale. Hence, we must accommodate the incremental transformation of health care from its current form toward an appropriately integrated and specified health care information system. This poses a substantial challenge with the current structure of health care information systems. The goal is to develop information support that can distill and represent relevant knowledge from health care processes and thereby support continuity and procedural interoperability (7).

According to the health care standards body HL7, FHIR resources support clinical workflow and activities performed or ordered by health care professionals to enable data interoperability between disparate health care entities who share patients (8). Such activities are defined within multiple terms that are built from semantic concepts with different facets and meta-data in multiple dimensions. Achieving workflow interoperability using FHIR requires standardization of processes, activities, and roles between heath care entities. The capability to support such planned sequencing of workflow and data exchange varies between entities according to the level of digital maturity of healthcare information systems. While a number of proposed solutions for care plan support have been published and implemented, there is to date no consistency between them and often a very sparse description of the informatics concepts.

Ambitious frameworks have been published such as the openEHR framework (9), the Cerner® Millenium framework (10) and the EPIC® FHIR based care plan (11). While these published frameworks have the ability to annotate plans, the created data still do not carry a full reference to the care plan structure and thereby miss part of the contextual information. The lack of overall orchestration of the adoption of these standards has led to a mixed landscape in which healthcare information systems are often not directly interoperable, which limits their applicability in care plan execution. Also, the systematic analysis of results is hampered as the differences also involve the semantic concept annotation that does not include a reference to the care plan and therefore limits the easy access methodology for real time analytics. The most important common limitation is that data acquired with the support of a care plan are annotated without the full vector content of the information (see below). Also, the somewhat artificial separation between the electronic medical record and the personal health record creates a difficulty because of the differences in legal framing and organization of these [for definition: (12)].

A workflow or care plan carries knowledge not only in the single action but also in the sequence of actions. In an ambitious attempt to increase the semantic precision of terms, efforts toward the creation of archetypes (13) and enriched concepts in SNOMED CT (14) have been made. These attempts showed certain promise at the time of development but the complexity and local (i.e., non-general) attempts to annotate the semantic relations between the partial concepts that constitute a SNOMED CT term has been met with limited adoption. The highly detailed relation between different partial concepts limits the intuitive use and also limits the interoperability between concepts and increases the number of concepts markedly. SNOMED CT today holds more than 120,000 unique codes and many more synonyms and abbreviations. In comparison the common human languages normally contain some 20,000 actively used words plus an extra 20,000 passive words. There are 2,539 terms in SNOMED CT related to diabetes with highly varying linguistic reach, semantic meaning and construction as well as categorical characteristics^1^ (15). This massive number of representations emphasize the effort to remove ambiguities in medical records but are clearly cumbersome and error prone as a tool in every-day clinical work. Furthermore, SNOMED CT terms are not accompanied by unambiguous definitions, such as explicit diagnostic criteria. This challenges the semantic interoperability of clinical concepts that are recorded in any two separate systems that both use SNOMED CT. The interoperability becomes heavily dependent on the (often not explicit) logic used by the local terminologists and clinical informaticians that labeled the source information. Given this potential for different clinical realities being encoded by the same SNOMED CT code due to local variation in information-labeling practices, it should come as no surprise that analysis of data originating from two or more such separate systems is vulnerable to bias. Moreover, the nature of the real-world processes underlying any large-scale efforts aiming to analyze data originating from multiple healthcare institutions (e.g., research consortia), is positioned to make this vulnerability wide-spread and common. Generic mapping tables, such as those published by the Observational Medical Outcomes Partnership (OMOP) or Unified Medical Language System (UMLS) are unable to account for local variation in coding practice. The development of bespoke mapping tables for use in individual systems is resource intensive and relies upon detailed knowledge of local information models and processes. There are no established quality standards for the development and validation of mapping tables with the ensuing risk of data distortion and systematic bias in cross system quality and outcomes research.

Sometimes health informatics seeks a level of exactness that is not found in natural language (16) where the domain of semantics is split between the words, their order in a sequence of words, flexibly used categorical hierarchies and also influences from other contextual domains. The effort to pack semantic content in a single term constrains the reach and leads to the splitting of concepts that in daily language are not separated into different archetypes. Thus, binding of clinical action to concepts becomes tedious and non-intuitive and therefore limits the usefulness in everyday work as it becomes necessary to involve experts to perform the work.

### Proposed Solution

To circumvent these vulnerabilities and prepare healthcare systems for the opportunity to execute standard clinical programs across multiple sites, we propose an alternative application of the care plan concept based on implementation of machine readable and machine executed support for consecutive health care activities. During the course of the project we developed a number of principles and building blocks all published in the public domain. Firstly, we explored the semantics of a multi-professional and patient centered process and the ability to allow for information support that intuitively could be understood by the users. We had the ambition that the work should be characterized by concepts from current developments in medical practice such as patient-centered care, shared decision making, value-based health care and team-based care.

Secondly, we defined an information model that allows the planning of data capture and the automatic extraction of individual data for analysis purposes. The information model supports the semantic needs that allow the definition of all necessary aspects for characterization of the data. The information model allowed a strategy for annotation of the data that fully reflects the care process.

**Figure 1:**
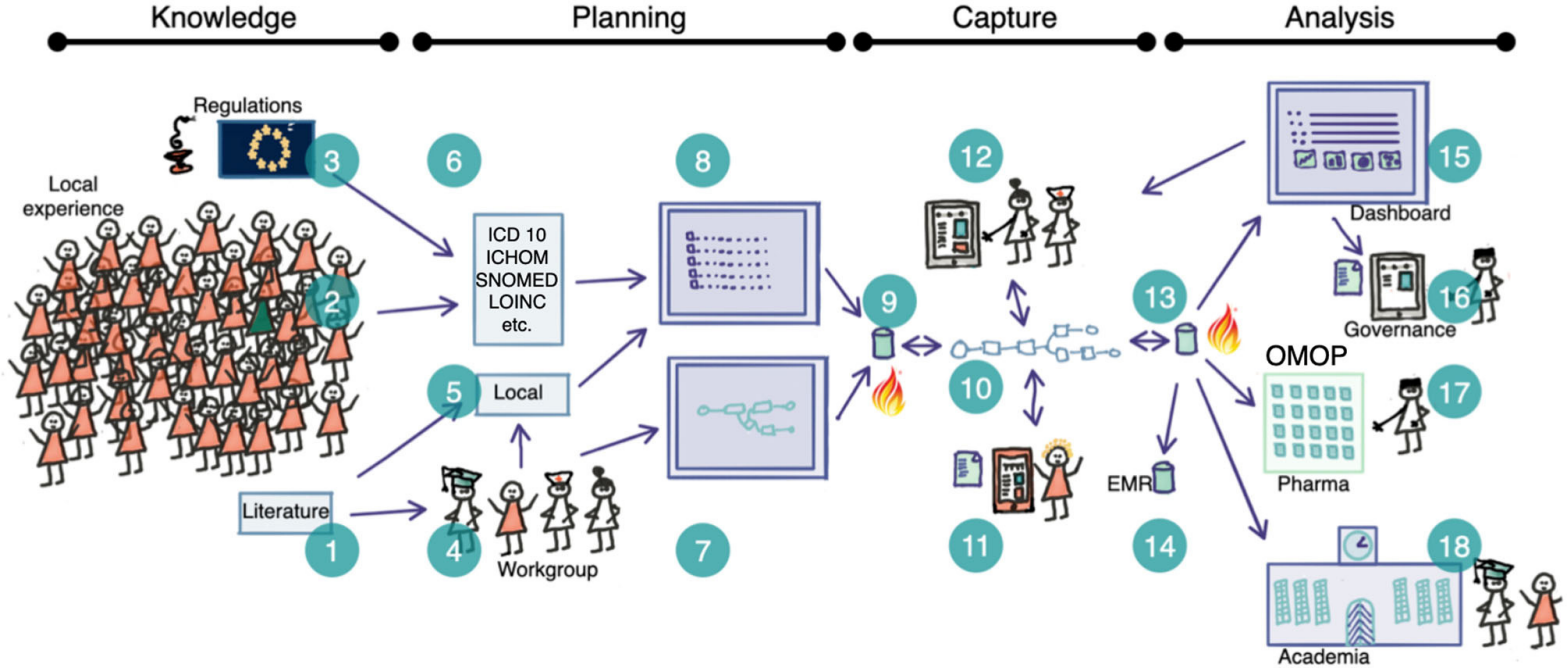
Schematic overview of the planning-capture-analysis steps where semantically competent care plans are used as a basis for clinical operational interoperability The idea is based on the ability to capture structured data where the full semantic depth is retained in all steps. Therefore we need complete standard meta-data that give the provenance of each term (1–3, 6) and the ability to add local terms (5). Clinical workgroups (4) create clinical pathways (7) that are populated with terms (8) and formatted into FHIR Plan Definitions and stored (9). Healthcare professionals and patients generate real-world data by documenting the routine execution of clinical processes at their local institution prompted according to the care plan (10) and collected by the clinical data capture tool (11, 12). All data are collected in the local database (13) and transferred to the EMR (14). The RWD functions allow for dashboarding (15) and feedback to care/patients as well as analysis by different stake holders including regulators, academia (18) and industry (17) (anonymised data).

Thirdly, we implemented in a case study all elements of the theory to test the developed construct. An overview of the strategy is given in Figure 1 and stipulates that the planning, capture and analysis steps should be separated but co-planned^2^. The case aimed for a proof of concept regarding the construction of care plans in the clinical interprofessional setting, the use of orchestrated structured data collection and finally to verify the functionality of the developed information model and the common data model and prepare for large scale implementation.

## Methods

### Design Considerations

When describing workflows, the information solution should be person-centric and ideally allow for the representation of relevant abstract concepts (Table 1). These abstract concepts ought to be delivered showing what are sequenced vs. not sequenced actions (i.e., chronological in order), planned vs. unplanned actions, while simultaneously being profession and care organization agnostic. It should also abide by conventions in clinical informatics concepts (17), prevailing standards, and secure data integrity (18). Another feature, often neglected, is that the more that the use of informatics principles mimic the structure of everyday language (without loss of precision), the more accessible it is for the practitioners.

**Table 1:** The different concepts from modern health care development that have been targeted to be met with the methodology presented in this paper. Their respective impact on informatics was determined by the authors for later inclusion in the construction of the present framework. References: Person-Centered Care (19), Interprofessional care (20), Value based healthcare (21). Shared decision making (2), Embedded trials (22).

**Theoretical framework**	**Semantic needs**
**Patient-centered care**	Transparent planning, Patient access to all information, Data obtained from the patient with annotation side by side in the medical record
**Interprofessional care**	Multi-tier care plan with transparent assignment of responsibilities, intuitive use of language
**Value based health care**	The ability to measure patient outcomes and PROMs across the full care cycle with fully planned terminology and procedures
**Shared decision making**	The ability to fully show detailed future alternatives for diagnosis and treatment and to involve the patient fully in all components of the decision making be it for big or small decisions.
**Embedded clinical RWD generation**	The generation of real-world data needs tools that generate fully semantically annotated but anonymous data

The concepts should be independent of the organizational structure and not lock procedures into the current organization of healthcare (visit types, profession specific activities, specific logistic procedures, etc.). Thus, in all components it should be based on a person centric aspect and all data that has been generated should be annotated to allow for full understanding of the semantic content of each data point (Figure 1: item 13). If this is achieved, multiple use of data (documentation, quality control, business intelligence, research, etc.) is possible without laborious mapping and post-*hoc* assignment of semantics. Each of these suggested uses of data has different requirements for the annotation and all aspects should be present in the information and annotation model. This minimizes the effort in extract, transform and load (ETL) procedures preparing for analysis as well as the storage of data (e.g., data-lake structures).

The ability to plan, execute and document a planned sequence of events in health care requires a richer data structure than conventional EMR systems typically provide, as the latter are dedicated mainly for documentation (Table 2). A care plan has components from multiple ontological domains that are normally not used in “documentation only” EMR systems. Such domains pertain to planning, logistics, assignments to health professions and individuals within the professional domain, reminders, warnings of deviation etc. A care plan is essentially a planned process and set of requirements subject to statistical process control (23) and quality. Hence, such requirements also enter into the specification of the annotation (24).

**Table 2:** The three principal methods of obtaining clinical data in ordered processes for trials, quality control, quality registries and as real-world data.

**Principles**	**Method**	**Pros and cons**
**EMR extraction** and post hoc reconstruction of sequence	Data extracted based on a manually determined index date and assigned meta information on the nature of the content	This requires manual mapping and post-assignment of semantic meaning to each data point which makes it less robust.
**Index date method** as used in most Case record file (CRF) methodology or quality registry protocols. Often parallel data collection outside the clinical process	An index date is set prospectively, and all data is annotated in the CRF or other repository with respect to the time-point. EMR is separate and used for reference only.	This method is robust but laborious. It necessitates a setup of a dedicated data-capture method often as a one-off construct (a CRF).
**Care plan** annotated data	Data is captured and annotated with the information on what care plan is used and the placement of the action in the care plan	The method that we have developed to continuously extract quality data, process data and real-world data from the clinical activity. Allows concomitant care plans that guide data capture for different purposes.

Extracting data from the EMR necessitates implicit semantic assumptions of the reasons of the actions that are made along the care trajectory. In the EMR case, the reasons for single actions and decisions are consecutively documented post-procedure by the professional responsible for the action, and often stored in free text format. This strips each collected data point of semantic meaning as there is not a point-to-point reference between the free text level and the points of measurements.

When automatic data extractions are made from the EMR, most often the timeline is used as a basis for post extraction semantic assumptions (see Table 2). The procedure for a blood pressure recording in the beginning of the care period is technically the same procedure as at the end of a care episode. However, the first one is probably used for diagnosis, or for general health evaluation, while the second may have completely different reasons such as screening for complications or side effects, detection of the effect of a drug, etc. Such differences are often accounted for by using different terms (concepts) for procedures, observations and intention in order to load each data point with semantic framing to allow retrospective understanding of the data. Consequently, SNOMED CT ends up with 200 concepts for blood pressure; a terminology with high level of granularity for each clinical concept but essentially unusable for practical purposes because of an abundance of specialized terms with limited applicability (e.g., SNOMED CT 718125007 Blood pressure cuff, reprocessed). Therefore, we saw the need to define a common information and annotation model that adheres to current standards whilst remaining easily implementable (usable) (Table 3) and concomitantly yields high precision while adhering to the prevailing standards in health informatics.

**Table 3:** An overview of the standards that the project considered in the development of a multipurpose care plan strategy.

**Domain of standards**	**Names of standards that have been addressed**	**Rationale**
Vocabularies	SNOMED CT, HL7 2, LOINC, ICHOM, ICD10/11	Vocabularies cover different ontological domains, and several are needed to build a care path. The local annotation with retained reference to provenance allows multiple standards to be used
Carepath support	OpenEHR, HL7 FHIR PlanDef/CarePlan	A number of standards exist. FHIR suited our purposes with its flexibility and growing adoption
Data reporting	FHIR Observation, FHIR Forms, OMOP, CDISC	FHIR observation is flexible, handles reference to care plan and readily translates into OMOP/CDISC
Representation of data collections	International Patient Summary, Medication list, Queries	These were broken down to term level and the components were represented in the local vocabulary allowing reconstruction to the protocols in the data reporting
Authentication	Smart on FHIR	Active monitoring of data communication protocols and security

The core instrument for holding medical information in health care is the electronic medical record (EMR) with the main function of supply non-changeable records as the basis for reimbursements and attribution of individual medical responsibilities. Thus, parts of the semantic content in the terms that are used are therefore lost once transferred to the EMR. In the current design, we therefore also considered how to perform programmed data collection as prescribed in quality registries and International Consortium for Health Outcome Measurements (25, 26). They have in common the logic of planned data collection and repeated measures obtained from the same individuals (before and after procedure or just cyclically repeated sampling in chronic conditions) which is the essence of data collection based on a care plan to build the evidence base (27).

A key issue that we addressed is the absence of a dominant standard for care plan derived data annotation that fully utilizes the semantic potential. The most elaborate framework to date is the HL7 FHIR based PlanDef, FHIR CarePlan and FHIR Observation standards. We therefore developed an information model and principles for annotation with reference to those resources.

### Key Developments

Ethical approval: Implementaiton of new work-processes and assisting technology and collection of data for national quality registers are not considered research in the meaning of the Swedish law regulating external ethical review. The observational study added to the clinical protocol was pre-approved by the Swedish Ethical Review Authority. All software that deals with patient data is subjected to the appropriate regulatory approvals including necessary level of CE label.

**Figure 2:**
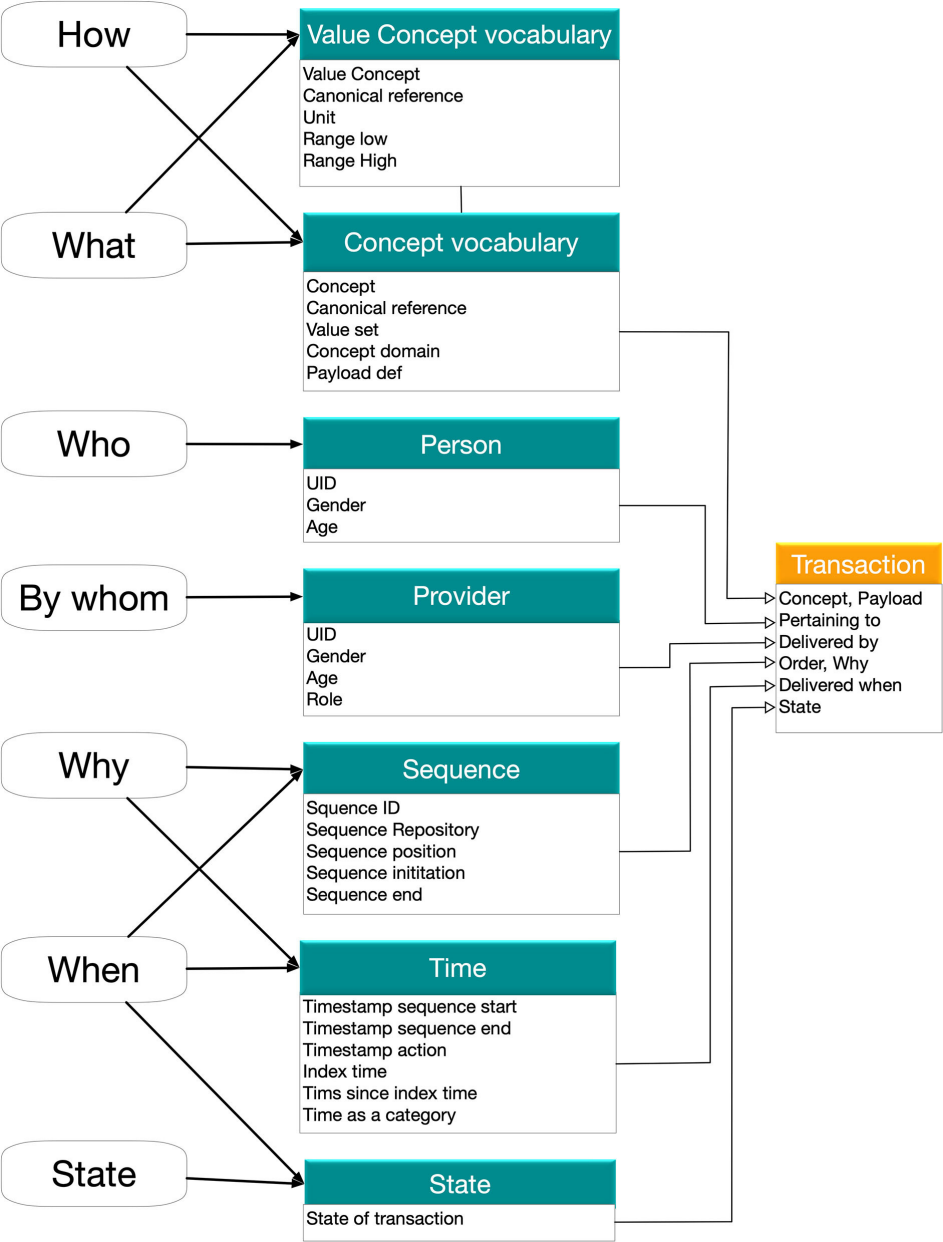
The information model for the single transaction that represents the lowest level of analysis. The necessary semantic dimensions are represented in two levels with the concept level (What/How/Who) and the sequencing level (By whom, why, when, state). All this can be represented in common standards such as FHIR Observation. The two-level model limits the need for the most complex SNOMED CT concepts (see text). A set of planned transactions with the state marked as planned and scheduling (times in the future) is the content of a careplan.

We used the strategy of limiting the semantic domains in each concept to only describe what the concept pertains to and, in case of procedures, reference to how it is performed (what and how). Semantic characteristics regarding aspects of intention, reason for procedure, sequencing and timing were all deferred to the care plan level (sequence, context, why, when, state). This two-level semantic annotation strategy still allows mapping of terms to standard vocabularies such as SNOMED CT and LOINC but only the most straight forward terms are deployed. The information model allows for a compact annotation of the identified semantic domains (Figure 2) and to minimize post-annotation of metadata. To fully characterize the use of a concept along the patient trajectory, the information model was developed with the patient trajectory as the core structure detailing it to the level of a transaction (activity). Thus, any transaction is related to a person (patient), has a payload of a defined concept, belongs to a sequence (care plan), is in a state (planned, in process, executed, forgotten, actively omitted), has a timing and is provided by a person directly or indirectly who belongs to a providing organization. This simple information model allows the construction of both a general data model and annotation model that fully account the care process and its underlying decision structure.

In order to retain the ability to use data for multiple purposes to serve patient needs, we placed the software for execution of care plans in between the patient and the EMR and secondarily mapped information objects for documentation according to the requirements of the local EMR system. This was a laborious and to some extent a sensitive process that needed governance. Also, the local law that prohibits the patient reported data to directly enter into the EMR (without the explicit endorsement of the clinician) had to be accounted for in the design of the procedures.

The developed procedure allows for the use of local terms based on local agreements in the work group (Figure 1: item 5) and could also be matched to standard vocabularies (Figure 1: item 6). The local terms were defined in a hierarchical structure and included version handling (Table 4) and were created and stored in a local simple data base tool that also could export the terms in lists that constitute the basis for a care plan (Figure 1: item 8). Also, the response options were listed as value concepts in the data base and grouped in value sets that could be bound to different terms. The term data base tool has been made available in the public domain (28) based on a commercial data base platform (Filemaker Pro 19, Claris International, Santa Clara, CA). The tool we developed is considerably more simple to use than existing and more ambitious public domain solutions (29). The structure of the term-database allows the non-expert to use and edit it when building local term repositories.

**Table 4:** The term construct that was developed. The term annotation was common for all three steps (planning, capture and analysis) and thereby any measurement or observation can be traced back to its definition.

**Term attribute**	**Description**	**Table in database**
TermUID	Unique identifier	Terms
Term name	Brief descriptive name	Terms
Term description	Descriptive text and possibly a reference	Terms
Canonic vocabulary	If available a reference to the concept in SNOMED CT, LOINC, ICD 10/11, ICHOM or other bodies for standardization of terminology	Terms
Canonic name	Name of term in reference vocabulary	Terms
Canonic version	Version of term in reference vocabulary	Terms
Canonic id	UID of term in reference vocabulary	Terms
Response classification	Kind of response requested (Categorical, number, date etc. …)	Terms
Response value set	A value set has multiple value concepts bound to it	List of value sets
Value concepts	Singular response concepts	Value concept list
Term list	A list of terms that provide a basis for the care plan	Term lists

### Annotation of Care Plans and Data Generated Based on Care Plans

The desired care path was defined on paper by the local care team (including patient representatives). In the patient centered view a visit to health care is a major event and within a visit there are a number of activities that may or may not lead to the generation of observational data. All activities are dispatched to different professional roles and may require sequencing, i.e. contain mandatory defined relations in the time domain with other activities. Standard annotations of the defined pathway in BPMN 2.0 (BPMN.org) is recommended, especially if the care pathway is shared between care providing units.

**Figure 3:**
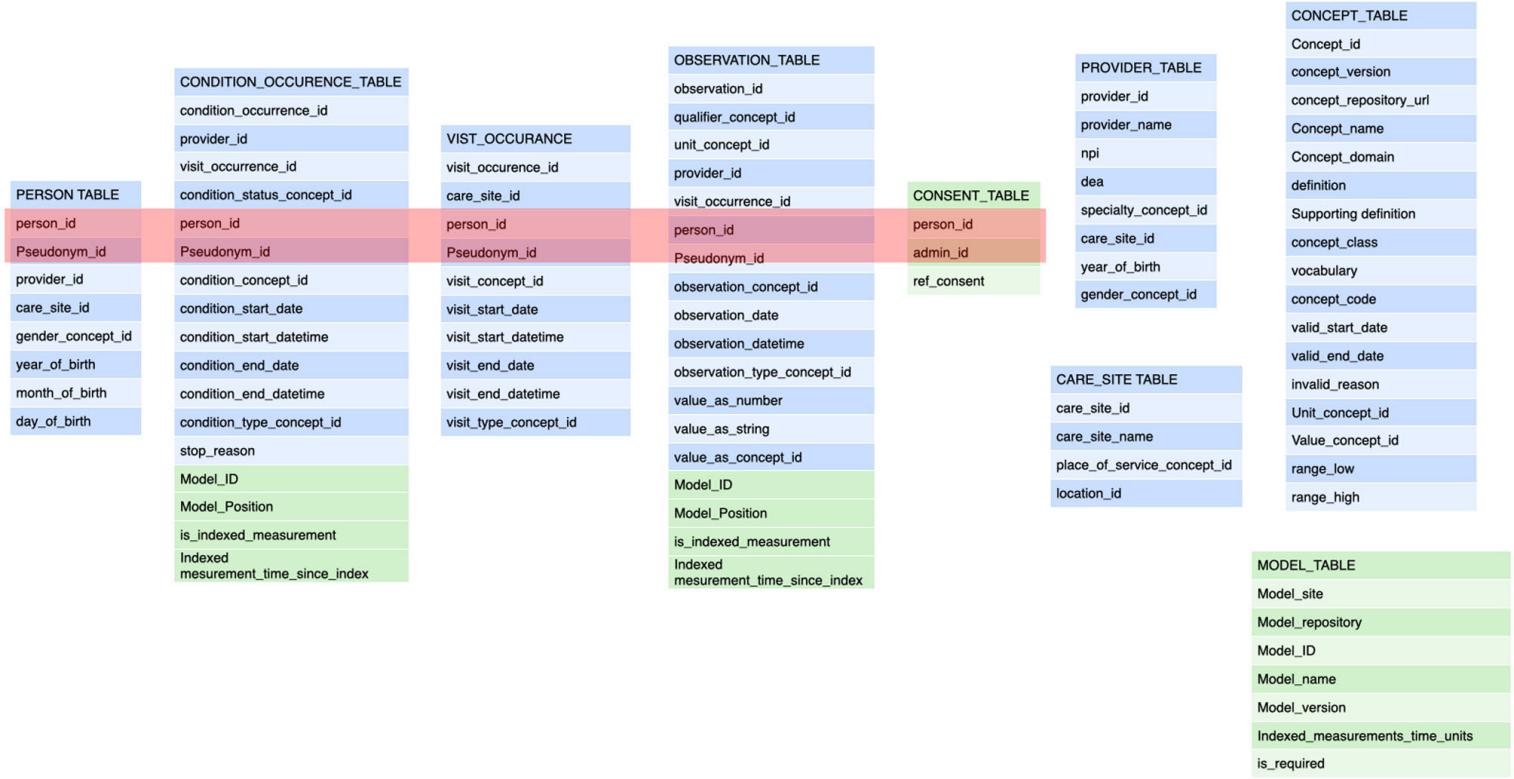
These are the tables for the **annotation** of a transaction. The tables refer back to the general annotation that is dictated by the information model (Figure 2). The tables adhere to the FHIR Observation resource and are derived from the OMOP data model (blue components). The modifications from the FHIR resources pertain to the collapsing of tables from FHIR observation, FHIR measurement, FHIR procedure to a single table as that implies semantic content that is determined at the transaction level. Also, the specific reference to the semantic level 2 (the care plan level) including the site in the plan is an important addition (depicted in green). This amendment allows the data to be fully anonymised by removing the links to a single person and the linking along the time domain. The data may still be used for e.g. outcomes estimations. See Figure 4. The embedding of clinical studies in clinical routine also necessitated the annotation of the informed consent status to properly manage the extraction of research data. It should be noted tha t this annotation carries both the information on the concept level as well as all necessary information on the sequence level (care plan level).

Within the concept of a FHIR PlanDef the order of **activities** is defined and the payload of one or more terms are identified for each activity. At the time of execution the PlanDef is instantiated as an individually assigned care plan (**FHIR CarePlan**). The execution of the individual patient CarePlan is guided by clinical process software (published elsewhere) that holds both the planning and guiding of the order of procedures. Each activity with its payload of terms generates one or more **FHIR observations or FHIR forms**. The FHIR observations are annotated according to the common data model for the project (Figure 3). The data model was derived to also satisfy the OMOP standard (30) which is designed to make data available for multiple purposes and has a solid translation table to FHIR resources such as FHIR Observation. The major addition (depicted in green) to the model was the explicit reference in each observation to the care plan_ID and the position in that care plan (Time as categorical concept). Also, the capability of the FHIR observation of annotating date/time elapsed from index date/time was used as this is a common standard for annotation in clinical development.

**Figure 4:**
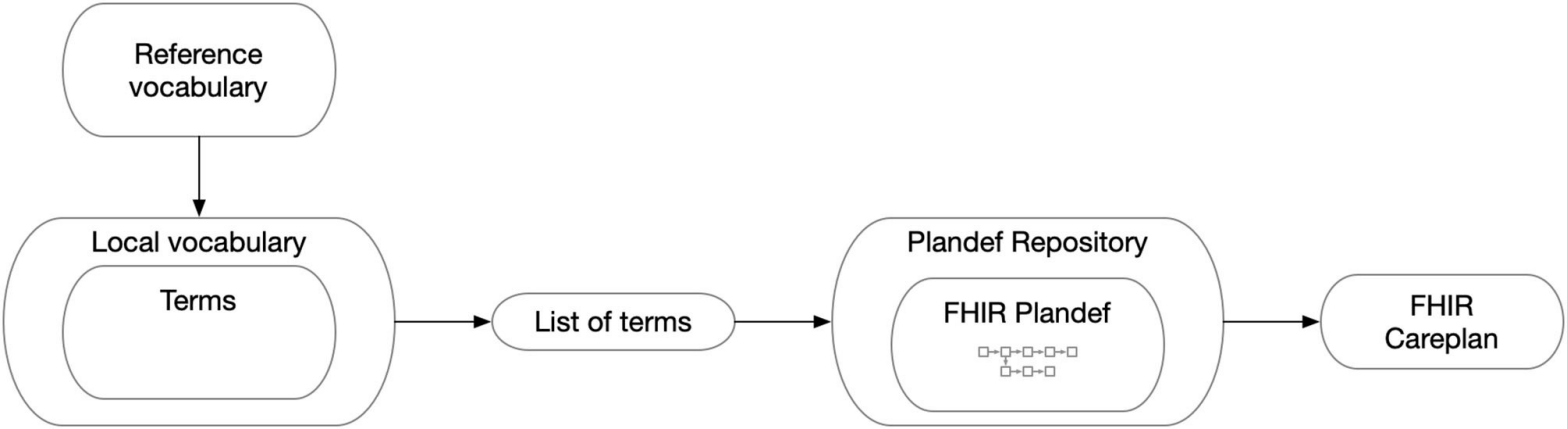
The general map of how the FHIR Careplan was constructed. Thus, FHIR observations generated on the basis of the individual care plan have information on vocabulary and its related value concepts as well as plan origin and placement in the plan. From this, all semantic information can be reconstructed for analytical purposes.

Thus, all observations have a reference to the applied care plan (child) which in turn has a reference to the PlanDef (parent) thus allowing complete and automatic reconstruction of the semantic properties underlying the observation. The full reference to the local repositories for the term and plan definition is given in separate tables and may accompany each data-point as needed. Thus, each observation has a full reference to all available semantic information regarding the clinical action of obtaining the observation (Figure 4). In the case where the data is conveyed to the EMR, these additional tables are dropped and data are stored according to the local EMR.

**Figure 5:**
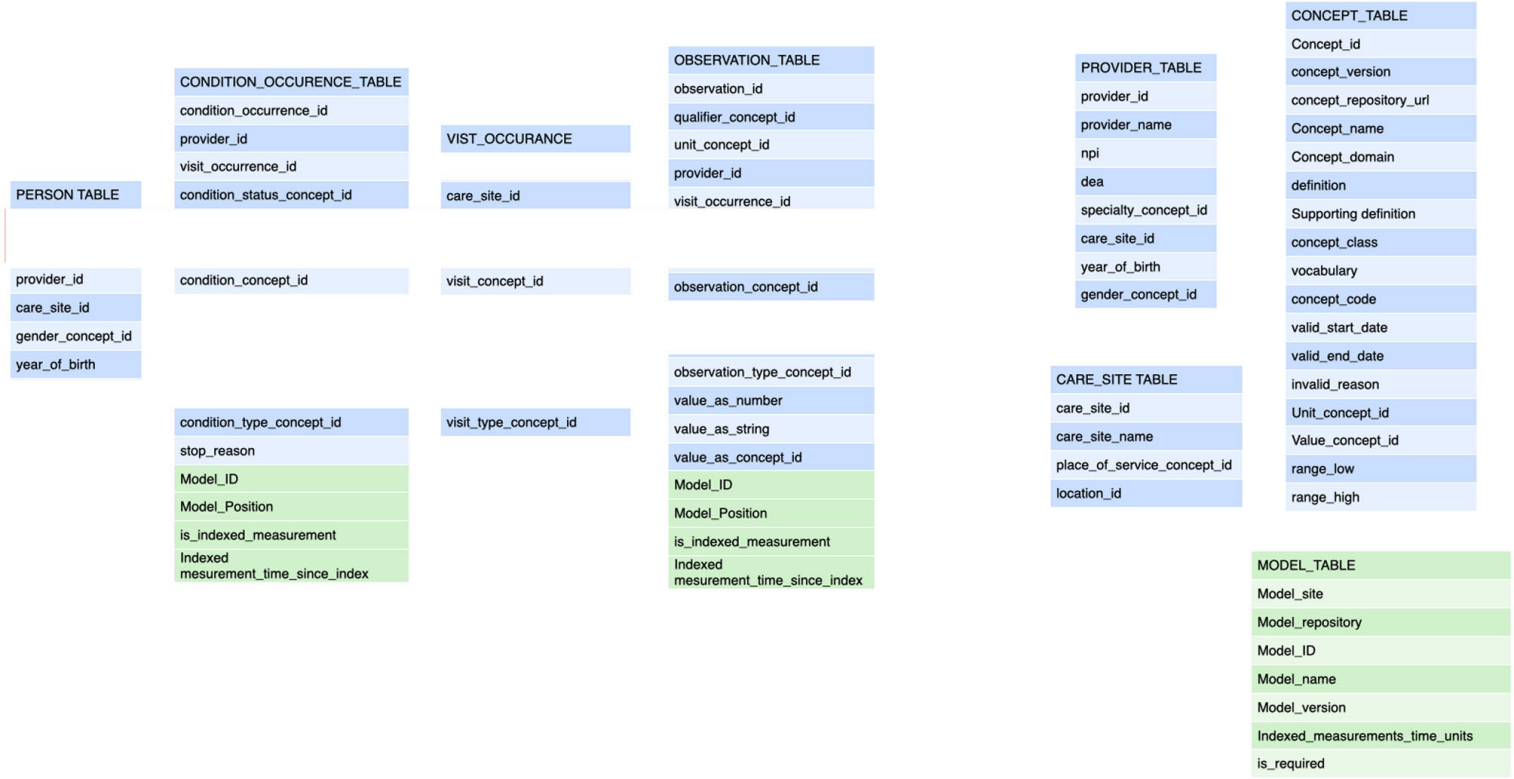
The data model provides the ability to fully anonymize the data by strict adherence to care plan annotation. All links pertaining to the patient ID and links across the continuous time domain are removed and replaced with a categorical time domain that adheres to the position in the care plan (Model Position).

The explicit reference to the care plan position of each action/observation allows for a very important addition to the concept. By republishing the data locally where all identifying data has been removed (name, pseudonym, case number, links in the time domain and reference time) data can be anonymized (Figure 5). Since the only link for each data point that remains is the link to the care plan no retrograde reconstruction of the identity of a subject can be made without access to the original data. Hence, data may be transformed from the status of individual data of sensitive nature that require all the provisions according to GDPR (31) to a status of fully anonymised thereby becoming non-sensitive data (GDPR terminology) for quality and research purposes.

In order to prepare for the case study we organized the clinical setting, prepared manuals etc. The procedure for process development was tested in a live clinical setting in a multi-professional group in charge of an out-patient specialist level clinic for medium to advanced stages of COPD (Figure 1: item 4, see also below case description). The representatives in the design were patients, doctors, nurses (including a research nurse), dietician, physiotherapist, occupational therapist, social worker and secretary. Each participant had the task of involving collegues in the analysis of the work content. An internal coordinator was responsible part-time for the development and the project was initiated from the clinical leadership level. The evaluation of the case study was made by collecting feedback during the development and use of the clinical tool, by inspection of the resulting Plandefintion and concepts, and finally by inspection of collected data that was generated for different purposes.

### Results: A Case Study of Initial Use of the Developed Framework

Prior to the start of this study, the clinical process support software had clinically been tested (>1,000 patients) in other clinical settings in a version with a less elaborate single careplan design. We tested the newly developed concepts within the realms of an out-patient, specialist level clinic for COPD patients (32) with the aim of scrutinizing the ability for care professionals themselves leading the process on the clinical level, the technical functionality of the developed concept and the viability of the work model before scaling for full clinical launch. The defined clinical plan that we developed was a work-up of a COPD patient following an exacerbation requiring hospital care with the aim to optimize treatment and follow up and thereby minimize the risk for new exacerbations. The program consists of a first meeting with full team evaluation, staging and an establishment of a treatment program. Follow-ups were scheduled individually as needed with a minimum of semi-yearly follow up. The clinical program stipulates that when in better shape the patients are referred back to primary care.

**The clinical professionals were able to devise the work model autonomously:** The list of terms to be employed in the clinical process was determined in an iterative approach where the concept needs for the clinical follow-up, the quality registry documentation and an observational design were determined separately in work-groups. The clinical protocol was given primary priority and the other two were harmonized with the clinical concepts (25) to minimize the burden of registration and to minimize visits to health care yet fulfilling the evidence-based monitoring needs as defined in the national guidelines for treatment of the patient group. The cyclic feedback from the different professions was used to modify the process to satisfy the perceived clinical needs. Initally, feedback after each patient was collected and later following small groups of patients. The feedback indicated that the process could be followed but that simplifications were warranted, Each iteration lead to further simplification and streamlining of the process. Two full scale iterations and several partial iterations were made. The collected information was also used for the planning of the clinical dashboard which was under construction.

**In all digitisation projects in clinical practice, to be successful, it is of major importance that the end-users are included in the development phase**. Considerable effort went into the education and motivation of the staff as the concept of guided data collection for the group level was not commonly understood. In the application clinic an interprofessional work model was already implemented. It represents a major transition to work with clinically guided comprehensive care-plans and this step is crucial for success. It is commonly known that interprofessional work-models can become ineffective due to the need of frequent meetings and often necessitate discussions on responsibilities. We sought to reduce the number for such meetings with the developed tool and the feedback where different dashboards were used for different professions and clear care-plan driven data collection was distributed across the professionals delivered the necessary clarity regarding the responsibilities. However, the transition toward knowledge sharing by data required instructional efforts. The construction of the care plan could surprisingly well be managed locally by a group of health professionals and patients based on a minimum of instructions. We had devised a manual for clinical development of care plans in self-managing workgroups consisting of different professions and patients that was available during the work.

Each profession developed their needs for terms, and required feedback on dashboards, in the design phase. Later, the removal of redundant data requirements and sequencing was authored in a combined group. It was noted that the work-group could internally agree on what should be done clinically (e.g., query smoking status) and together construct a preliminary term list (e.g., use standard form from quality register) in order to monitor patients with a set diagnosis with a minimum of instructions. The preliminary list was then annotated in the local concept repository including its unique identifier, descriptive name, definition, reference to a canonical concept (if available), the chosen value set with its value concepts, units, cardinalities and defined tolerances.

**A reduced set of non-complex terms could be used to support the clinical process**. The selected terms were bound, by external expertise and when considered appropriate, to canonical vocabularies (e.g., SNOMED CT, ICD10) and thereafter mapped to the annotation system of the local EMR (Cerner, Melior). The clinical activities of COPD resulted in the identification of 540 unique terms including those where the patient responded directly to questions in the waiting room before the visit to the team. Some 60% were bound to canonical vocabularies whereas the rest were designated as part of the local vocabulary. Only a small minority of responses (<3%) were defined as free text response. Matching of the response items was also performed to support concomitant data collection to the National Airway Quality Registry (Figure 1: item 16). Also, for exploratory purposes, we included terms from an observational study beyond what was needed for optimal clinical handling in order to test the concept of clinically embedded observational studies. The case record file (CRF) was built separately and then amalgamated in the main term collection. Duplicates were removed and adjustment of terms were performed to harmonize the semantics of terms and still allow use of the data for different purposes (25). The introduction of the observational study added 199 terms to the list.

The final clinical protocol contained 739 terms covering the domains that support the continuous development of the evidence base (27). Data were transferred to the local EMR system at the end of each action following a display of a summary and a sign-off from the health care professional. The intent was to minimize redundant documentation and simplify the record-keeping. Considerable further effort will be needed to fully reach this goal.

When comparing the quality registry term base with the term list many of the registry terms were already in the term list, though adjustments had to be made on the local level. The lack of harmonization of requested data from the county level quality assessment strategy and the national level quality registry created a delicate problem and, in some instances forced double registration of overlapping but not identical observations. The burden of registering data for the national quality registry are expected to be significantly reduced once the full implementation is made.

We added a clinical observation study protocol as a stress test for the concept. Firstly, the protocol was constructed as a standalone CRF and reformatted according to the care pathway format. Then it was merged on the care plan level and terms were adjusted as much as possible to correspond to the clinical work group selection of terms and response options. Hence, a single term used in the encounter with a patient could result in data that could be used for all three purposes directly.

**Initial clinical testing showed the desired functionality:** The data capture along the patient centered process was successful and initial evaluation showed that the data was of sufficient quality to satisfy all three purposes (clinical, quality register and observational study). All professionals and the patient added information along the route. In several iterations the process defining the patient trajectory was redesigned and simplified. The conclusion was that the clinical support system was ready to be used on scale in clinical routine for all three identified purposes. Quantitative results from the implementation is the next step.

## Discussion

We give an account for the results of a methodological development and the initial practical steps to share the efforts that are necessary to technically support the change of working patterns in health care from an iterative manual approach (expert-apprentice model for knowledge transfer) to a planned process approach (industrial model for knowledge transfer). Such processes need to be developed in order to handle the societal pressure on the health care sector to be more person oriented (33), eliminate waste (5), and to have the ability to continuously report on the quality in terms of delivered patient value according to the principles of value based health care (21). Chronic disorders and multimorbidity continue to increase in prevalence (1) and by this the demand for patient centered effective continuity in care and decision-making increases. Also, multi-morbidity yields an increased complexity and often a multi-professional team approach. When a number of health care professionals work in collaboration, effective collaboration tools need to be developed to minimize the loss of effectivity with manual work and communication practices.

The major implications and recommendations for practice are discussed below.

1) The key professions need to be involved in the transformation of work processes.

Moving from the current iterative decision making in healthcare to planned patient trajectories is a major change in the way care is organized. As in all organizational changes the inertia to change has many origins. The strict distribution of medical responsibility in health care key professions is a major factor that conserves the pattern of work and the principles of documentation of the work. There is therefore a strict need to involve the professions in the transformation. Hence, we made considerable effort to provide tools for the care professionals to participate. The split of the semantic annotation between the ***what***, and ***how*** properties that were tied to the concept level and the ***why***, ***when*** properties that were tied to the care plan level was very well received in the clinical context as the clinical work-groups that were able to work on quite advanced process modeling with full understanding of all used concepts.

2) The terminology for clinical action should be based on a limited size vocabulary with a limit of the semantic complexity in each term in order to support participation of local health care professionals.

When the clinical work is supported by full care pathways or snippets of such pathways procedural interoperability is supported. However, the buy in of the key professions on the design of such support is central. We noted that adherence to protocol and the joy in work fell dramatically when the relevance of procedures was considered obscure. The cultural capital of the health care staff in always delivering patient safety and relevant care must be regarded as an important asset (34) that should be safe-guarded and nurtured by the change in work procedures.

3) The information solution should promote procedural interoperability.

Procedural interoperability means the decisions should have low variability between health care professionals and support a shared structure of the decision tree. Serving the right knowledge at the right time is crucial in order to effectively serve the needs of the patient. While data availability is very good in healthcare the data presentation and the derivation of knowledge from the data is far less evolved (35). It is both a matter of a lack of data aggregation but also simple user experience (UX) principles on accessibility, consistency in presentation and user controllability are often not implemented. As each patient with chronic disease has an abundance of data created along the disease trajectory the ability to uphold the principle that all decisions should be based on all relevant data is hard to reach.

4) The information architecture must change from being provider oriented to be person centered.

Noting that the aim of the EMR is primarily to safeguard the professional and economic goals of the organization, the support for continuity and procedural interoperability becomes a secondary goal. The latest generations of major EMR systems all contain some sort of care pathway support for execution but the annotation of data in general does not support the full semantic content in the way it has been described here. Thus, in order to create meaningful group level feedback post annotation assignment of semantics needs to be applied in order to achieve e.g. the simplest of dashboards. In contrast, the design we demonstrate here allows an annotation of the desired semantic aspects to each data point and the use of data for different purposes such as the EMR, patient communication, dashboards, RWD and clinical studies is easily attainable. The radically patient centered model does not preclude the derivation of the necessary provider-oriented data such as records of medical decision making and recording of information with economic bearing. In fact, when tested, the continuous extraction of such data is made easier by the data annotation principles that we present.

5) The information architecture should promote the rapid cycle feedback to health care in order to support procedural interoperability.

The developed framework allowed us to go further in the automatic derivation of knowledge from data as the local professionals all can influence how clinical feedback should be formed on both the individual and group levels. As noted above, the annotation of the data creates a direct opportunity to extract group level data for direct feedback to the clinical environment. Thus, the information circle that promotes the concepts of learning health care is supported in real time. A major weakness in current quality registry architecture is that most do not support such direct data use as they often are subject to late reporting and sparse cyclic feedback. The concept that we have presented allows for real time feedback and repeated analysis by a standard algorithm. The behavioral effects of the rapid feedback is now possible to assess in a quantitative way. This will be the subject of a coming publication.

The architecture is also ideal for use of machine learning for identification of deviations from planned events but also to identify redundant data collection. The proposed standardization of the annotation of the recorded observations with the associated meta-data makes this concept very adaptable to the requirements in FAIR principles (36) and newly developed concepts for distributed analytics according to the concepts of “The Personal Health Train” (37).

The ambition of our multi-year project was to challenge the inertia for change that many have identified in health care. In the seminal book on value based health care, Porter and Teisberg identified the resistance to change in health care stemming from the non-alignment of stakeholder goals (21). In this communication we have identified the prevailing structure of the health information systems as an added factor to health care system inertia to change. The Porter/Teisberg book introduced the concept of created patient value that health care has produced as a means to align the stakeholders. At the same time, it was clear that the assessment of patient value was a theoretical construct but there was no real standard of how to obtain sufficient data. With the inception of ICHOM it required a decade to establish international standards for the measurements of patient outcomes. The systematic implementation of the ICHOM measures in healthcare has been hindered by the lack of patient centered health information systems with proper semantic models. The collection of data that reflect clinical outcomes and patient outcomes requires an information system that prompts patients and health professionals to capture data at scheduled time points across the full care-cycle. To date, this work is mostly organized with make-shift solutions and often entails manual checklist or similar. This lack of standardization, also delays the feedback to patients and health care of the acquired knowledge and therefore diminish the power to promote a systems level change. The concepts presented in this paper are such that they allow for gradual adoption and thereby one of the sources of the inertia to change is mitigated.

## Conclusion

This clinically experimental project is based on the identified need for information supporting technology that is compatible with the gradual change of health care to become more person centric and allow support of health professionals in their work and to allow multiple data use for science and quality control along with the record keeping. We have designed and tested a number of concepts and find that unless the information model is flexible in supporting the use of clinical pathways in an accessible way, with methods where the professionals are part of the construction, system level inertia from professional roles, administration systems, payment systems and poor information technology will prevent health care development. To that end we needed to develop additions to prevailing data annotation models in order to fully use the vector properties of data captured based on care plans.

## Data Availability Statement

The original contributions presented in the study are included in the article/supplementary material, further inquiries can be directed to the corresponding author/s.

## Author Contributions

All authors have contributed to parts of the design and assessment of results of the study as well as read, suggested alterations, and approved the manuscript. Other significant contributions MI, CW, and SH: concept. MI, CW, and LV: execution. MI: initial writing.

## Funding

MI received support from The Swedish Innovation Agency Vinnova for part of this work. LV received support from The Family Kamprad Foundation, The Swedish Heart and Lung Foundation and ALF VGR. The funding agencies had no influence on the content of this communication. Part of the work presented in this paper draws on knowledge, insights, experiences in Gravitate-Health, funded under IMI2 JU grant agreement No 945334.

## Conflict of Interest

MI is a co-founder and member of the board of the not-for-profit ICHOM.org and GR is a current employee of ICHOM. The testing software for care plans was commercially developed by an SME, Frisq AB (publ). The authors declare that all components pertaining to the formation of the term sets and components of the common data model are published to the public domain. The testing software for care plans was commercially developed by an SME, Frisq AB (publ). CW has worked as a consultant and core developer of the software. MI and CW have assigned the conceptual IP regarding planned data capture to FRISQ in 2017 in order to fund further development. MI and CW has a minimal ownership in FRISQ (<0.3 %). The remaining authors declare that the research was conducted in the absence of any commercial or financial relationships that could be construed as a potential conflict of interest.

## Publisher's Note

All claims expressed in this article are solely those of the authors and do not necessarily represent those of their affiliated organizations, or those of the publisher, the editors and the reviewers. Any product that may be evaluated in this article, or claim that may be made by its manufacturer, is not guaranteed or endorsed by the publisher.

## Footnotes

^1^A quote from a team member during the development of this work: “Writing a medical note becomes like writing a novel in a foreign language and only have a dictionary of full sentences at hand.”

^2^In Figure 1, step 10 the actual execution of the care plan was guided by a purpose-built CE labeled transaction engine specifically constructed for the purpose (https://www.frisqcare.com). Plan Definitions were loaded manually (will be automated in the future) directly into the tool and guide both the care and the patient along the health trajectory. The tool supports interprofessional collaboration around the patient trajectory and guides the necessary data collection. The output of data is mapped onto the EMR but also supplies necessary data to a quality register and clinically embedded studies. The reason for using a purpose-built tool is that the care plan implementations in current EMR systems do not allow the necessary annotation for data intended for multiple purposes nor do they support the desired interprofessional collaboration.
